# Sex, gender and immunosenescence: a key to understand the different lifespan between men and women?

**DOI:** 10.1186/1742-4933-10-20

**Published:** 2013-05-16

**Authors:** Calogero Caruso, Giulia Accardi, Claudia Virruso, Giuseppina Candore

**Affiliations:** 1 Department of Pathobiology and Medical and Forensic Biotechnologies, University of Palermo, Corso Tukory 211, Palermo 90134, Italy

**Keywords:** Sex, Gender, Ageing, Immunosenescence

## 

Gender and sex are known to be associated with longevity. While males are usually stronger, females live longer. In the Western world, the life expectancy of individual born between 2005 and 2010 is 80.4 for women and 73.4 for men [1]. Potential factors have been examined to explain this disagreement. It is possible distinguish advantage in longevity related to biological traits and factors related to socio-cultural characteristics of the population. Males and females have different behavioral tendencies, social responsibilities and expectation. So, differences in mortality between men and women can be not only a matter of sex that refers to biological differences, but also a matter of “socially constructed sex”, i.e. gender [2,3].

One of the main interaction between gender and longevity is linked to the kind of job. Indeed, in the to-day elderly, professional exposure to stressors was stronger in males rather than in females [4]. Since many classic “old” jobs needed specific physical characteristics, females were often housewives, hence they were commonly more protected. Furthermore, alcoholism, smoking and accidents were the main factors contributing to excess male mortality although it is’nt anymore true in the actual generation [2,3]. On the other hand, it is well known that female mithocondria produce less reactive oxygen species than male ones and that estrogens increase high density lipoproteins and decrease low density ones [5,6]. Thus, both gender and sex might be responsible for the differences in lifespan between males and females [2,3].

However, immune-inflammatory responses play a key role in successful ageing [2]. So, immunosenescence, a complex process in which different immunological functions are impaired, others are remodeled, is believed to be a major contributory factor to the increased frequency of morbidity and mortality among elderly [2,7]. On the other hand, it is still controversial whether age-related changes of immune system are different between men and women.

To elucidate the relationship between immunological changes and lifespan, peripheral blood mononuclear cells of 356 healthy Japanese ranging in age from 20 to 90 years were analyzed for the number and percentage of various lymphocytes by using three color flow cytometry [8]. The proliferative and cytokine producing ability of T cells in response to anti-CD3 monoclonal antibody stimulation was also assessed. The results show that an age-related decline is observed in the numbers of T cells, in certain subpopulations of T cells (including CD8+ T cells, CD4+CDRA+ T cells, and CD8+CD28+ T cells), and B cells, and in the proliferative ability of T cells. The rate of decline in these immunological parameters, except for the number of CD8+ T cells, is greater in male than in female. An age-related increase is observed in the number of CD4+ T cells, CD4+CDRO+ T cells, and NK (CD56+CD16+) cells and in the CD4+ T cell/CD8+ T cell ratio. The rate of increase of these immunological parameters is greater in female than in male. The T cell proliferation index (TCPI), which was calculated based on T cell proliferative activity and the number of T cells, shows an age-related decline. The rate of decline in the TCPI is again greater in male than in female. T cell immune score, which was calculated by using 5 T cells parameters, also declines with age, and the rate of decline is greater in male than female. In addition, a trend of age-related decrease was observed in the production of some cytokines, when lymphocytes were cultured in the presence of anti-CD3 monoclonal antibody stimulation. In particular, the rate of decline in IL-6 and IL-10 is greater in male than in female [8]. Because IL-10 acts as an immune-inflammatory suppressor [9], this relatively lesser production can be consistent with the fact that the age-related decline of various immunological parameters is less pronounced in female than in male.

The authors conclude that the age-related changes of various immunological parameters is different between men and women, likely due to a lower biological age of women. These findings, indicating a slower rate of decline in these immunological parameters in women than in men, are consistent with the fact that women live longer than men, i.e. in Japan 85.5 years in women and 79.0 in men [8].

It is indeed well known that the strength and the kind of immune responses are different between males and females. Hormonal and genetic influences are the main biological differences to consider when the attention is focused on immunology. While from a gender point of view food intake and variety, exposure to non-microbiological antigens and health care access have to be take into account [2,10].

Steroid hormones, linking to specific receptors, modulate in different manner the immunological cells. Estrogen receptors have been detected not only in classical reproductive tissues, but also in immune cell population, including lymphocytes, monocytes and macrophages [2]. In general, while estrogens action increase the immune response, it falls with progesterone and androgens action [10]. As an example, estradiol activates the mitogen-activated protein kinase (MAPK) pathway that leads to the downstream activation of nuclear factor kappa B (NFkB) signaling pathway. Both, MAPK and NFkB pathways, are involved in enhanced expression of genes involved in immune response and in genes encoding antioxidant enzymes [11].

A sexual dimorphism in the immune response means that females are more resistant to infections but they have higher incidence of autoimmune diseases compared to male [12], but their relevance for life span is negligible [13].

In addition to hormones, the most intuitive genetic factor that can determine difference in the immune response between male and female is the X chromosome, since it is well known that some genes involved in immunity map in this chromosome. However, other important genes are located on autosomes although they are regulated in sex-specific manner. Since X chromosome is present only in one copy in male, every X chromosome random recessive mutation will be expressed. It is not the same for female in which two copies are present at tissue level (“mosaicism”) balancing the mutation [3,10,14-16].

Another cellular process that differs between male and female and that can play a role, is the rate at which telomeres shorten since women have less telomere shortening than do men. However, telomere shortening may be a cause for and/or a consequence of immunosenescence [17].

On the other hand, whereas sexual differences can advantage females, gender differences can damage them. In fact, financial trouble and cultural factors are the cause of a reduced consumption of food for female. Indeed, they are often more prone to the renounce thus they are mainly subjected to malnutrition. Food intake and composition can modulate the immune response trough the lack of micronutrients and vitamins, essential for immune cells. Vitamins affect mast cells function and immunoglobulin, NK and lymphocyte number [18]. The lack of zinc and copper, immunomodulatory micronutrients, can, also, negatively affect the immune response in gender specific manner [19,20]. In term of health care, then, females are underprivileged while males and children have often the priority. Thus, for example, female have less access to antibiotics and chemioterapics [21]. These gender differences might explain why in certain developing countries the female life expectancy is almost similar to male life expectancy [1].

Finally, it is well known that men and women follow different trajectories to reach longevity. The reasons are most likely multifactorial, involving genetic, epigenetic and environmental factors [22]. Several key molecules and regulatory pathways have been identified that may play a role in determining lifespan and new molecular mechanisms that regulate longevity, are waiting for to be uncovered. The detection of potentially involved mechanisms might allow the way to a better identification of anti-aging strategies.

## Competing interests

The authors declare that they have no competing interests.

## Authors’ contributions

CV drafted the paper. All authors edited the paper and approved its final version.
